# Trachomatous Growths upon the Vocal Cords

**Published:** 1885-04

**Authors:** A. W. Calhoun

**Affiliations:** Professor of Diseases of the Eye, Ear and Throat in the Atlanta Medical College, Atlanta, Georgia


### TRACHOMATOUS GROWTHS UPON THE VOCAL CORDS.

### A CLINICAL LECTURE.

By A. W. CALHOUN, M. D.,

Professor of Diseases of the Eye, Ear and Throat in the Atlanta Medical College, Atlanta, Georgia.

Reported for THE ATLANTA MEDICAL AND SURGICAL JOURNAL.

The three cases that I now present to you, gentlemen, are suffering from trachomatous growths upon the vocal cords, and are exceedingly rare.

Out of several thousand cases examined by me with the laryngeal mirror, I have only found five cases suffering from these growths. One of these five cases was partially relieved by operation, that will be described to you in a few minutes ; but I lost sight of the case, and do not now know how it terminated, not having seen or heard from him in more than three years. Another one of the five cases was entirely cured and remains so, he still being under my observation.

Two of the cases were men, one sixty-five years old, the other forty years old; one a boy—the case you now see—thirteen years old; the other two cases we show you are two little girls, six and eight years of age. The boy has several large growths upon both vocal cords, growing from their under surface. One of these growths is found just between the anterior ends of the cords.

The effects of these growths are apparent, as you perceive in this patient. He has lost his voice; his breathing is extremely difficult. His mother says his breathing at night is truly alarming.

It seems as if he could not live through the night. She is in constant dread of his dying.

These trachomatous growths are generally caused by some excoriation of the mucous membrane of the vocal cords. Hot air inhaled, steam, or anything that tends to cause abrasions of the vocal cords may produce the disease. Sometimes, however, they seem to appear without any known cause.

In this boy's case we find the cause to have been the inhalation of steam from an overturned boiler. The trouble set up some weeks after this accident and has been constantly growing until now it is with difficulty that he breathes or talks.

When these growths are small and do not produce much inconvenience, even though they may affect the voice, it is better to let them alone; but if they are increasing in size, as they are in the case before you, it is only a matter of time, and not a very long time either, when the larynx will become entirely closed, and death will be the result. In all such cases an operation is the only thing that promises relief to the patient.

You cannot make the operation without the aid of the laryngoscope. With the throat well lighted up and the tongue drawn forward, with the laryngeal mirror the growth is perfectly displayed, then with a pair of McKenzie's cutting forceps, such as I now show you, you clip off as much of the growth as your patient will stand. The larynx soon becomes so sensitive that you cannot continue the operation very long at one sitting, but by repeated operations you will succeed in getting away the entire growth or growths. I have already made several operations on this boy, removing small portions each time. The mass is soft, and is not difficult to remove when you get your forceps on it.

After entire removal of these trachomatous growths there is very little tendency to recurrence.

In the case of the little girl the growth is so large, and she is so young and unmanageable, that I fear I will be compelled to make a thyrotomy, that is, cut through the thyroid cartilage directly upon the vocal cords, and remove the growth *en masse*. It is a very rare and dangerous disease. The operation on the little girl will be deferred until another day.

